# Causal Effects of Serum Levels of n-3 or n-6 Polyunsaturated Fatty Acids on Coronary Artery Disease: Mendelian Randomization Study

**DOI:** 10.3390/nu13051490

**Published:** 2021-04-28

**Authors:** Sehoon Park, Soojin Lee, Yaerim Kim, Yeonhee Lee, Min Woo Kang, Kwangsoo Kim, Yong Chul Kim, Seung Seok Han, Hajeong Lee, Jung Pyo Lee, Kwon Wook Joo, Chun Soo Lim, Yon Su Kim, Dong Ki Kim

**Affiliations:** 1 Department of Biomedical Sciences, Seoul National University College of Medicine, Seoul 03080, Korea; mailofsehoon@gmail.com (S.P.); yonsukim@snu.ac.kr (Y.S.K.); 2 Department of Internal Medicine, Armed Forces Capital Hospital, Seongnam 13574, Gyeonggi-do, Korea; 3 Division of Nephrology, Department of Internal Medicine, Uijeongbu Eulji University Medical Center, Uijeongbu 11759, Gyeonggi-do, Korea; sjlee891016@hanmail.net (S.L.); wooo35@empas.com (Y.L.); 4 Department of Internal Medicine, Keimyung University School of Medicine, Daegu 42601, Korea; yaerim86@gmail.com; 5 Department of Internal Medicine, Seoul National University Hospital, Seoul 03080, Korea; ahdia0602@naver.com (M.W.K.); imyongkim@gmail.com (Y.C.K.); hansway80@gmail.com (S.S.H.); mdhjlee@gmail.com (H.L.); junephro@gmail.com (K.W.J.); 6 Transdisciplinary Department of Medicine & Advanced Technology, Seoul National University Hospital, Seoul 03080, Korea; kksoo716@gmail.com; 7 Department of Internal Medicine, Seoul National University College of Medicine, 101 Daehak-ro, Jongno-gu, Seoul 03080, Korea; nephrolee@gmail.com (J.P.L.); cslimjy@snu.ac.kr (C.S.L.); 8 Kidney Research Institute, Seoul National University, Seoul 03080, Korea; 9 Department of Internal Medicine, Seoul National University Boramae Medical Center, Seoul 07061, Korea

**Keywords:** coronary artery disease, mendelian randomization, polyunsaturated fatty acids, myocardial infarction, risk factor

## Abstract

We aimed to investigate the causal effects of n-3 and n-6 polyunsaturated fatty acids (PUFAs) on the risk of coronary artery disease (CAD) through Mendelian randomization (MR) analysis. This MR study utilized a genetic instrument developed from previous genome-wide association studies for various serum n-3 and n-6 PUFA levels. First, we calculated the allele scores for genetic predisposition of PUFAs in individuals of European ancestry in the UK Biobank data (*N* = 337,129). The allele score-based MR was obtained by regressing the allele scores to CAD risks. Second, summary-level MR was performed with the CARDIoGRAMplusC4D data for CAD (*N* = 184,305). Higher genetically predicted eicosapentaenoic acid and dihomo-gamma-linolenic acid levels were significantly associated with a lower risk of CAD both in the allele-score-based and summary-level MR analyses. Higher allele scores for linoleic acid level were significantly associated with lower CAD risks, and in the summary-level MR, the causal estimates by the pleiotropy-robust MR methods also indicated that higher linoleic acid levels cause a lower risk of CAD. Arachidonic acid showed significant causal estimates for a higher risk of CAD. This study supports the causal effects of certain n-3 and n-6 PUFA types on the risk of CAD.

## 1. Introduction

Coronary artery disease (CAD) is a comorbidity that critically affects patient prognosis and is associated with a substantial socioeconomic burden globally [1]. A major goal of current medical interventions for metabolic disorders is to prevent CAD, but CAD is predicted to remain the primary cause of death worldwide along with obesity and global aging trends. Thus, identifying protective or causative factors for CAD is an important health issue that may suggest preventive measures for CAD development.

As metabolic disorders are common predisposing factors for CAD [2], maintaining a healthy diet has been suggested to be an important lifestyle modification strategy for preventing CAD [3]. Among the dietary components, dietary fat intake is one of the factors affecting CAD development, and controlling dyslipidemia is important for the primary and secondary prevention of CAD. Substituting saturated fats with unsaturated fats has been recommended in the American College of Cardiology/American Heart Association and US dietary guidelines and has shown benefits in reducing CAD risks in clinical studies [4,5,6,7,8]. Among the unsaturated fats, polyunsaturated fatty acids (PUFAs), particularly n-3 and n-6 PUFAs, have been emphasized for their possible significant effect on the risk of cardiovascular diseases [4,8]. However, the observed findings reported different CAD risks according to the PUFA subtypes [9] and are inevitably prone to be affected by confounders or reverse causation. Thus, additional studies identifying the causal effects of various n-3 or n-6 PUFAs on the risk of CAD are warranted. However, although such evidence was presented by the previous GISSI trials for secondary prevention after the development of heart failure or coronary artery disease by n-3 PUFA supplementation [10,11], whether increasing PUFA intake may be helpful for primary prevention of CAD remains controversial due to heterogeneous intervention and the results of previous trials [12,13].

Mendelian randomization (MR) is a useful tool for investigating causal effects from modifiable exposure to complex diseases [14]. In MR, the exposure of interest is explained by genetic instruments, and as one’s genotype is determined upon conception preceding the occurrence of confounders or diseases, MR can report causal estimates minimally affected by confounding effects or reverse causation. MR has been implemented in the medical literature and has identified important causal factors for the risk of CAD or other chronic comorbidities [15,16,17,18].

In this study, we aimed to investigate the causal effects of serum n-3 and n-6 PUFA levels on the risk of CAD by MR analysis testing the association between genetic predisposition for each PUFA type and the risk of CAD or myocardial infarction (MI). We performed both allele-score-based MR by individual-level data and MR based on summary-level data in different cohorts to replicate the findings. We hypothesized that certain n-3 or n-6 PUFAs would have causal effects on the risk of CAD.

## 2. Materials and Methods

### 2.1. Study Setting

**Figure 1:**
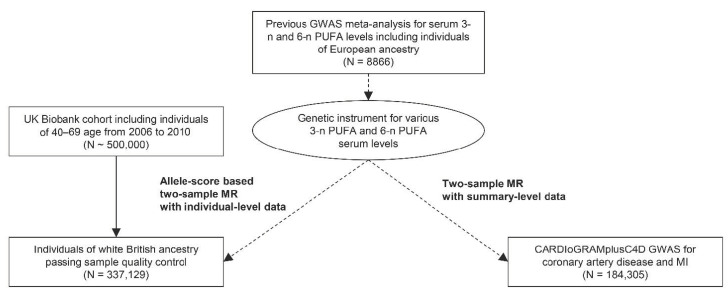
**Study flow diagram**. The study consisted of two parts; a two-sample MR analysis based on summary-level data with the CARDIoGRAMplusC4D data and an allele-score two-sample MR analysis based on individual-level data the UK Biobank data. GWAS = genome-wide association study; MI = myocardial infarction; PUFA = polyunsaturated fatty acid.

This study was an MR analysis including genetically explained exposures and outcomes from observational cohorts independent of the population where the genetic instrument was developed (i.e., two-sample MR). The study first utilized the UK Biobank data, which is the largest cohort to date with deep genotyping and collection of various clinicodemographic information [19]. The UK Biobank data have been introduced as the outcome data for allele-score based MR by the individual-level data. In addition, we performed summary-level MR with another independent observational genome-wide association study by the CARDIoGRAMplusC4D consortium. The analysis was performed to ask whether our findings can be replicated by another large-scale cohort by MR based on summary statistics (Figure 1).

### 2.2. Genetic Instruments

The study utilized two well-known genome-wide association meta-analysis results for the serum levels of specific types of n-3 and n-6 PUFAs of individuals of European ancestry [20,21]. The study included genome-wide significant (*P* < 5 × 10^−8^) single nucleotide polymorphisms (SNPs) that were not in linkage disequilibrium (*R*^2^ < 0.1) with the PUFAs identified by the GWAS. The genetic instruments were repetitively utilized in the literature, including MR analysis, to study the causal effects of PUFA levels on various diseases and were identified to be on genes that are relevant to lipid metabolism [22,23,24]. We utilized the genetic instruments for n-3 PUFAs (docosapentaenoic acid (3 SNPs), eicosapentaenoic acid (2 SNPs), and docosahexaenoic acid (1 SNP)) and for n-6 PUFAs [linoleic acid (3 SNPs), gamma-linolenic acid (2 SNPs), dihomo-gamma-linolenic acid (2 SNPs), adrenic acid (1 SNP), and arachidonic acid (2 SNPs)], and their summary statistics are presented in Table 1.

The MR investigation requires three assumptions to be met to demonstrate causal effects [14]. First, the relevance assumption means that the genetic instrument should be closely associated with the exposure of interest. As all included genetic variants reached genome-wide significance level association with each PUFA level and the variants were in functionally relevant genes for PUFA metabolism, the assumption was met. Second, the independence assumption indicates that the genetic instrument should not be associated with confounders or the absence of directional pleiotropy. The third assumption is the exclusion-restriction assumption, meaning that the causal effect should be only through the exposure of interest. The utilized genetic instrument for each PUFA phenotype included 1 to 3 SNPs with known functional relevance, which may decrease the possibility of heterogeneity, and the causal estimates by the instruments would be considered specific to the exposure of interest. In allele-score-based MR, we adjusted major clinical covariates to attain the independence assumption. In addition, we performed pleiotropy-robust MR sensitivity analysis in our summary-level MR investigation, which relaxes the second and third assumptions.

### 2.3. Allele-Score Based MR with Individual-Level Data in the UK Biobank

The UK Biobank is a prospective population-based cohort of >500,000 individuals aged 40–69 years from 2006 to 2010 in the United Kingdom. The details of the database have been published before [19,25,26]. For genetic analysis, as the genetic instruments were developed in individuals of European ancestry, we included the UK Biobank data of individuals of white British ancestry. We excluded those who were outliers in terms of heterozygosity or missing rate, those with sex chromosome aneuploidy, and unrelated samples who were included in the genetic principal component calculations [27]. The approach resulted in 337,129 individuals included in the genetic analysis with the UK Biobank data.

In the allele-score-based MR, we assessed the risk of MI in the UK Biobank data, which was algorithmically defined by the UK Biobank and included death from MI and ST-segment elevated MI or non-ST-segment MI events based on hospital admission records and death registries. We included events through 29 February 2016, as complete hospital inpatient data were available until that date in all three regions of the nation: England, Scotland, and Wales, in the current data.

We calculated allele scores for the exposures by multiplying the gene dosage matrix with the effect sizes of the genetic instrument by using PLINK 2.0 (version alpha 2.3) [28]. The associations between the genetic predisposition for each serum PUFA level represented by the allele scores and MI were investigated by logistic regression analysis, and age, sex, and the first 10 principal components were adjusted [16]. We additionally performed a sensitivity analysis by adding phenotypical hypertension, diabetes mellitus, obesity, medication use for dyslipidemia, laboratory values of triglycerides, low-density lipoprotein, high-density lipoprotein and smoking history (none, ex-smoker, and current smoker) to the regression model. The regression analyses were performed using R (version 4.0.1, the R foundation), and two-sided *p*-values < 0.05 were considered significant.

### 2.4. Summary-Level MR with the CARDIoGRAMplusC4D Data

Additional summary level-based MR was performed with the summary statistics provided by the CARDIoGRAMplusC4D study including participants mainly of European ancestry [29]. We tested the causal estimates for the MI (43,676 cases and 128,199 controls) and CAD (60,801 cases and 123,504 controls) outcomes from the CARDIoGRAMplusC4D GWAS results. The fixed-effects inverse variance weighted method was the main MR method. When the number of SNPs was 3, for linoleic acid, additional sensitivity analysis by the penalized weighted median method [30], which gives valid causal estimates even when invalid instrument is present, and by MR-Egger regression with bootstrapped standard error [31], which yields pleiotropy-robust causal estimates, were performed. When a single SNP was included in a genetic instrument, the causal estimates were driven by the Wald ratio method. The above analyses were performed with the TwoSampleMR package in R [32].

## 3. Results

### 3.1. Clinical Characteristics of the UK Biobank Data

The baseline characteristics of the UK Biobank participants of white British ancestry utilized for the genetic analysis are presented in Table 2. The median age was 58 years old, with 54% males and 46% females. The prevalence of hypertension and diabetes was 21% and 5%, respectively, with 18% of participants taking medication for dyslipidemia. The interquartile ranges for triglycerides, low-density lipoprotein, high-density lipoprotein, and estimated glomerular filtration rate values were within the reference ranges. The prevalent/incident MI outcome was identified in 12,812 (4%) individuals.

### 3.2. Allele-Score Based MR Results with the UK Biobank Data

The causal estimates by the allele score-based MR are presented in Table 3. Among the n-3 PUFAs, genetically predicted eicosapentaenoic acid levels were significantly associated with lower odds for MI, while the allele score for docosapentaenoic acid was significantly associated with higher MI risks. Genetically predicted docosahexaenoic acid showed null causal estimates. Genetic predispositions for higher linoleic acid and dihomo-gamma-linolenic acid were significantly associated with lower risks of MI. On the other hand, genetically predicted gamma-linolenic acid and arachidonic acid levels were significantly associated with higher MI risks. Adrenic acid showed null causal estimates for the risk of MI. The above results were similarly reproduced even after we included additional phenotypical covariates in the regression analysis.

### 3.3. Summary-Level MR Results with the CARDIoGRAMplusC4D Data

When the analysis was replicated with the CARDIoGRAMplusC4D data, the genetic predisposition for higher serum eicosapentaenoic acid levels was significantly associated with a lower risk of CAD, but not with the risk of MI (Table 4). For docosapentaenoic acid, the direction of the ORs was consistent with the above allele score-based MR; however, the causal estimates did not reach the statistically significant level. For n-6 PUFAs, higher levels of genetically predicted linoleic acid were marginally associated with a lower risk of CAD or MI by the inverse variance-weighted method. When additional pleiotropy-robust methods were implemented, both the penalized weighted median and the MR-Egger regression results indicated that genetic predisposition for higher linoleic acid levels was significantly associated with lower risk of both CAD and MI. The causal estimates were similar to the findings in the UK Biobank data for dihomo-gamma-linolenic acid and arachidonic acid, as genetically explained dihomo-gamma-linolenic acid was significantly associated with both lower CAD and MI risks, while arachidonic acid was causally linked to higher risks of both CAD and MI. Gamma-linoleic acid, in which the causal estimates were significant in the allele score-based MR, showed marginally significant causal estimates with notably high odds ratios in the summary-level MR, whereas adrenic acid, which showed null findings in the allele score-based MR, showed significant causal estimates for higher risks of both CAD and MI in the CARDIoGRAMplusC4D data.

## 4. Discussion

This study, including MR investigation, identified that serum levels of certain n-3 or n-6 PUFAs causally affect the risk of CAD or MI. Higher genetically predicted eicosapentaenoic acid was significantly associated with a lower risk of MI in the UK Biobank data and of CAD in the CARDIoGRAMplusC4D data. Dihomo-gamma-linolenic acid levels were significantly associated with a lower risk of CAD or MI. Linoleic acid was also considered to be protective against CAD or MI in our results. Arachidonic acid was the n-6 PUFA that showed significant causal estimates for a higher risk of CAD or MI. Higher docosapentaenoic acid and adrenic acid may also be causative for CAD development, but the causal estimates were inconsistent or marginal in our investigations.

The clinical significance of n-3 and n-6 PUFAs has been debated. Several observational findings and meta-analyses reported the possible benefits of n-3 or n-6 PUFAs on the risk of cardiovascular disease [4,8]. In addition, studies focusing on mechanisms of the protective effect of n-3 and n-6 PUFAs reported that PUFAs are related to atherogenesis, thrombotic activity, and inflammation [33,34]. However, there were also contradictory reports addressing the absence of an effect of n-3 or n-6 PUFAs on CAD [12]. Because observational findings can be affected from unmeasured confounding effects or reverse causation, causal interpretation of the previous observational studies is limited. Moreover, as the effects from specific PUFA types may vary, a clinical trial, which would reveal the causality of a PUFA on CAD, with a strict dietary modification for a single PUFA is difficult to perform, resulting in heterogeneous findings from previous trials [12]. Thus, whether higher serum PUFA levels or levels of a specific PUFA can be effective for primary prevention of CAD has remained unanswered.

We performed this study to investigate the causal effects of various PUFA types on the risk of CAD or MI by implementing MR analysis. MR has a particular strength in that the method can reveal causal estimates from a modifiable exposure to complex diseases [14]. The approach has now been widely introduced in the current medical literature and has reported causal effects from various exposures on complex diseases, which is difficult with randomized clinical trials. One’s genetic information is determined before the occurrence of any confounding factors or outcomes and is thus minimally biased by the effects from other clinical factors. In our MR analysis, we made efforts to attain the three key assumptions for an MR to demonstrate the causal effects. As the results were consistent for certain PUFA types, our study supports that serum PUFA levels causally affect the risk of CAD and MI. Moreover, our study reported that not all PUFA types uniformly reduce or increase the risk of CAD [35], and specific n-3 or n-6 PUFAs showed different causal directions. Thus, the findings may guide future trials that may prioritize specific PUFA types as interventional targets.

Among the most abundant n-3 PUFAs, our MR findings indicated that higher serum eicosapentaenoic acid may causally decrease the risk of CAD, while the results for docosapentaenoic acid were inconsistent. Primarily in liver, a partial conversion of n-3 PUFAs occurs by elongation and desaturation enzymes, converting α-linoleic acid to eicosapentaenoic acid, to docosapentaenoic acid, and to docosahexaenoic acid. A meta-analysis of 13 trials suggested that supplementation of marine n-3 PUFAs lowers the risk of CAD [36]. A previous report suggested that eicosapentaenoic acid may have a greater protective effect against CAD than docosapentaenoic acid. Additionally, supplementation with eicosapentaenoic acid has been reported to be preventive for CAD in hypercholesterolemic patients [37]. Recently, although a supplementation of marine n-3 PUFAs, including both eicosapentaenoic acid and docosapentaenoic acid, was not efficient to reduce the risk of cardiovascular disease [38], supplementation of purified eicosapentaenoic acid reduced adverse cardiovascular events in patients with high triglyceride levels [39]. Thus, eicosapentaenoic acid may be the prioritized n-3 PUFA as a supplemental target for the primary prevention of CAD. Regarding n-6 PUFAs, conversion from linoleic acid to arachidonic acid is more efficient than that of n-3 PUFAs. Among the n-6 PUFAs, linoleic acid has been repeatedly reported for its importance in the risk of CAD, and experimental findings support its benefits on the cardiovascular system [4,9,40]. The allele score-based MR and the pleiotropy-robust weighted median or MR-Egger analyses yielded significant causal estimates of linoleic acid on lower risk of CAD, which is consistent with the previous observational findings. A previous MR study reported null effects of linoleic acid on ischemic heart disease by utilizing different outcome summary statistics [24]; however, as genetic instruments explain only a portion of the variance of an exposure, the results warranted additional validation. As genetic predisposition for higher linoleic acid was significantly associated with CAD or MI risks both in the CARDIoGRAMplusC4D and the UK Biobank data, the causal effects from linoleic acid on CAD would be interpreted to be present, supported by previous observational findings. In addition, for dihomo-gamma-linolenic acid, previous clinical and experimental studies reported that high levels of dihomo-gamma-linolenic acid may be protective against cardiovascular diseases [35,41]. That an increased arachidonic acid level was causative for increased CAD or MI risks was also in concordance with previous observations reporting its proinflammatory role [42,43], and our study suggests the causality of its effect on the primary development of CAD. On the other hand, increased adrenic acid or gamma-linolenic acid serum levels were suspected to cause CAD or MI in the MR results [44]; however, as the results were inconsistent in the replicative investigation, a future study is necessary to confirm their significance.

Some of the MR analysis results need additional explanation. First, the statistical power of an MR investigation generally increases by utilizing a large number of genetic variants to explain the exposure of interest [45]. However, the currently available genetic instruments for each PUFA type included 1 to 3 SNPs, which may have low statistical power to capture the causal effects from genetic predispositions for PUFA levels. Particularly, that genetically predicted eicosapentaenoic acid being nonsignificantly associated with the risk of MI in the CARDIoGRAMplusC4D may be from that the prevalence of MI was lower than that of CAD in the data, thus, MI outcome being more affected by the possibility of weak instrument bias. Additional investigations including a larger number of independent variants to genetically explain the serum PUFA levels may clearly distinguish the risk of CAD by an MR investigation. However, utilizing a few SNPs would have reduced the possibility of horizontal pleiotropy which would bias the MR causal estimates thus, the positive findings identified in this study could be considered as actual causal effects of the genetic predisposition for each PUFA type on CAD risks. Next, as the actual size of an effect for relevant clinical intervention is different from the causal estimates in MR, the current MR results are qualitative information on the presence of causal effects from certain serum PUFA levels on CAD [46,47]. Namely, one genetically predicted serum PUFA showing a larger effect size than another does not mean that the first serum PUFA would have larger clinical effects than the second. That the prevalence of CAD or MI was different between the UK Biobank and the CARDIoGRAMplusC4D data also explains the considerable differences in the sizes of the causal estimates by the two separate MR analyses. Additional clinical trials targeting different n-3 or n-6 PUFA subtypes are warranted to confirm whether the suggested causal effects from PUFAs on CAD development can be modified through interventions for PUFAs and to identify the clinical magnitude of the effects.

There are several limitations and unanswered questions in this study. First, as stated above, although this study suggested the causal effects of certain serum PUFA levels on CAD development, whether an effective intervention of modifying serum PUFA levels can actually be helpful for primary prevention of CAD should be answered by a future clinical trial. Second, additional experimental study is warranted to reveal the mechanism of different effects of certain serum PUFA levels on CAD risks. Third, as MR is weak for detecting nonlinear effects and as quantitative interpretation of our results is limited, the extent to which a serum PUFA level is beneficial or harmful cannot be answered by this study. Last, the study was mainly based on individuals of European ancestry due to data availability, so our results cannot be generalized to other ethnic populations. Future development of large-scale genetic data including various ethnic populations would be helpful to expand the diversity of ethnic populations which can be investigated by an MR analysis.

## 5. Conclusions

In conclusion, this study supports the causal effect of certain n-3 and n-6 PUFAs on the risk of CAD or MI. Additional clinical trials targeting specific n-3 or n-6 PUFAs are warranted to reveal possible beneficial dietary interventions for the primary prevention of CAD and to determine the target population.

## Figures and Tables

**Table 1:** Summary statistics of the genetic instruments.

Phenotype	SNP	Effect Allele	Other Allele	Effect Allele Frequency	Beta	Standard Error
Eicosapentaenoic acid	rs3798713	C	G	0.43	0.035	0.005
	rs174538	A	G	0.72	0.083	0.005
Docosapentaenoic acid	rs780094	T	C	0.41	0.017	0.003
	rs3734398	T	C	0.43	0.04	0.003
	rs174547	T	C	0.67	0.075	0.003
Docosahexaenoic acid	rs2236212	C	G	0.57	0.113	0.014
Linoleic Acid	rs10740118	G	C	0.56	0.2484	0.0431
	rs174547	C	T	0.32	1.4737	0.0417
	rs16966952	A	G	0.31	0.3512	0.0439
Gamma-linolenic acid	rs174547	T	C	0.67	0.0156	0.0009
	rs16966952	G	A	0.69	0.0061	0.0009
Dihomo-gamma-linolenic acid	rs174547	C	T	0.33	0.355	0.0136
	rs16966952	G	A	0.69	0.22	0.013
Arachidonic acid	rs174547	T	C	0.68	1.6909	0.0253
	rs16966952	G	A	0.69	0.1989	0.0314
Adrenic acid	rs174547	T	C	0.67	0.0483	0.0019

**Table 2:** Baseline characteristics of the study population of the UK Biobank for the genetic analysis.

Characteristics	Total (*N* = 337,129)	Males (*N* = 156,106)	Females (*N* = 181,023)
Age (years)	58 [51; 63]	59 [51; 64]	58 [51; 63]
Body mass index	26.7 [24.1; 29.8]	27.3 [25.0; 30.0]	26.1 [23.4; 29.6]
Obesity (body mass index ≥30 kg/m^2^)	81,022 (24.1%)	39,328 (25.3%)	41,694 (23.1%)
Smoking history			
Non-smoker	183,636 (55%)	76,356 (49%)	107,280 (60%)
Ex-smoker	118,399 (35%)	60,835 (39%)	57,564 (32%)
Current-smoker	33,921 (10%)	18,360 (12%)	15,561 (9%)
Hypertension	70,018 (20.9%)	38,538 (24.9%)	31,480 (17.5%)
Systolic BP (mmHg)	136.5 [125.0; 149.5]	139.5 [129.0; 152.0]	133.5 [121.5; 147.5]
Diastolic BP (mmHg)	82.0 [75.5; 89.0]	84.0 [77.5; 90.5]	80.0 [73.5; 87.0]
Diabetes mellitus	16,178 (5%)	10,012 (6%)	6166 (3%)
Hemoglobin A1c (mmol/L)	35.1 [32.7; 37.7]	35.2 [32.7; 37.9]	35.1 [32.7; 37.6]
Medications for dyslipidemia	58,531 (18%)	35,832 (23%)	22,699 (12.6%)
Triglycerides (mmol/L)	1.5 [1.1; 2.2]	1.7 [1.2; 2.5]	1.3 [1.0; 1.9]
High-density lipoprotein (mmol/L)	1.4 [1.2; 1.7]	1.2 [1.1; 1.5]	1.6 [1.3; 1.8]
Low-density lipoprotein (mmol/L)	3.5 [3.0; 4.1]	3.5 [2.9; 4.1]	3.6 [3.0; 4.2]
Aspartate aminotransferase (U/L)	20.2 [15.4; 27.4]	23.8 [18.4; 31.8]	17.5 [13.9; 23.0]
Alanine aminotransferase (U/L)	24.4 [21.0; 28.8]	26.1 [22.6; 30.9]	23.0 [20.0; 26.8]
Creatinine (mmol/L)	70.5 [61.6; 81.0]	80.0 [72.6; 88.3]	63.2 [57.1; 70.0]
Estimated glomerular filtration rate (mL/min/1.73 m^2^)	92.5 [82.6; 99.5]	92.2 [82.6; 99.3]	92.9 [82.6; 99.8]
Number of prevalent/incident MI cases	12,812 (4%)	9878 (6%)	2934 (2%)

Categorical variables are presented as number (%) and continuous variables are presented as median [interquartile ranges].

**Table 3:** Allele-score based Mendelian randomization results in the UK Biobank data for MI outcome.

Genetically Predicted PUFA Level by Allele–Scores (1 Standard Deviation Increase)	Main Analysis ^a^		Sensitivity Analysis Adjusted for Phenotypical Covariates ^b^	
	Adjusted OR (95% CI)	*P*	Adjusted OR (95% CI)	*P*
n-3 PUFAs				
Eicosapentaenoic acid	0.973 (0.956–0.991)	0.003	0.969 (0.949–0.989)	0.002
Docosapentaenoic acid	1.027 (1.009–1.046)	0.004	1.029 (1.008–1.050)	0.006
Docosahexaenoic acid	1.000 (0.982–1.018)	0.986	1.003 (0.982–1.023)	0.804
n-6 PUFAs				
Linoleic acid	0.975 (0.957–0.992)	0.005	0.967 (0.947–0.987)	0.001
Gamma-linolenic acid	1.022 (1.003–1.040)	0.020	1.028 (1.007–1.049)	0.009
Dihomo-gamma-linolenic acid	0.972 (0.955–0.990)	0.002	0.969 (0.950–0.989)	0.003
Arachidonic acid	1.027 (1.009–1.046)	0.004	1.034 (1.013–1.056)	0.001
Adrenic acid	1.004 (0.986–1.022)	0.672	1.008 (0.987–1.029)	0.458

PUFA = polyunsaturated fatty acids; OR = odds ratio; CI = confidence interval; MI = myocardial infarction. All allele scores were scaled to a one standard deivation increase. ^a^ The logistic regression model was adjusted for age, sex, and the first 10 principal components of the genetic information. ^b^ The phenotypical hypertension, diabetes mellitus, obesity, dyslipidemia medication history, smoking, laboratory values for low-density lipoprotein, high-density lipoprotein, and triglycerides were added to the main model.

**Table 4:** Summary-level Mendelian randomization results with the CARDIoGRAMplusC4D data.

Genetically Predicted PUFA Level	For Coronary Artery Disease		For Myocardial Infarction	
	OR (95% CI)	*P*	OR (95% CI)	*P*
n-3 PUFAs				
Eicosapentaenoic acid	0.781 (0.626–0.975)	0.029	0.793 (0.537–1.172)	0.245
Docosapentaenoic acid ^a^	1.215 (0.971–1.522)	0.089	1.227 (0.954–1.578)	0.110
Docosahexaenoic acid	1.000 (0.851–1.175)	0.999	1.057 (0.883–1.264)	0.548
n-6 PUFAs				
Linoleic acid	0.987 (0.975–1.000)	0.055	0.986 (0.972–1.000)	0.053
Penalised weighted median ^b^	0.986 (0.974–0.999)	0.035	0.984 (0.970–0.999)	0.033
MR-Egger ^b^	0.979 (0.958–1.000)	0.024	0.975 (0.951–0.999)	0.022
Gamma-linolenic acid	2.541 (0.783–8.244)	0.120	2.960 (0.790–11.097)	0.107
Dihomo-gamma-linolenic acid	0.940 (0.897–0.985)	0.010	0.932 (0.884–0.983)	0.009
Arachidonic acid	1.012 (1.000–1.024)	0.042	1.014 (1.001–1.027)	0.037
Adrenic acid ^a^	1.587 (1.054–2.391)	0.027	1.700 (1.073–2.693)	0.024

PUFA = polyunsaturated fatty acid; OR = odds ratio; CI = confidence interval. ^a^ The causal estimates were driven by the Wald ratio method. Otherwise, the fixed effects inverse variance weighted method was implemented. ^b^ Additional sensitivity analyses were performed as 3 SNPs were included in the genetic instrument for linoleic acid.

## Data Availability

The data described in the manuscript will be made available from the UK Biobank consortium after acquiring approval (URL: https://biobank.ctsu.ox.ac.uk/crystal/docs.cgi?id=1, last accessed on 22 April 2021). The code book and analytic code will be made available by the corresponding author upon reasonable request.
